# Comparison of Sleep Features Across Smartphone Sensors, Actigraphy, and Diaries Among Young Adults: Longitudinal Observational Study

**DOI:** 10.2196/67455

**Published:** 2025-08-14

**Authors:** Jaclyn S Kirshenbaum, Ryann N Crowley, Melissa D Latham, David Pagliaccio, Randy P Auerbach, Nicholas B Allen

**Affiliations:** 1 Department of Psychiatry, Columbia University, 1051 Riverside Drive, Pardes 2313, New York, NY, 10032, United States, 1 6467745376; 2 Department of Psychology, University of Oregon, Eugene, OR, 97403, United States; 3 Ksana Health Inc, Eugene, OR, 97403, United States; 4 VA Northern California Health Care System, Mather, CA, 95655, United States; 5 Division of Child and Adolescent Psychiatry, New York State Psychiatric Institute, New York, NY, 10032, United States

**Keywords:** mobile sensors, accelerometer, wearables, daily diary, sleep measurement

## Abstract

**Background:**

Poor sleep health is pervasive and contributes to long-lasting physical and psychological problems. As traditional sleep measurement can be burdensome, testing scalable and accessible sleep measurements is important.

**Objective:**

The aim of this study was to test whether sleep features obtained through a smartphone app are comparable to other modes of sleep measurement (ie, daily diary and wearable actigraphy).

**Methods:**

Healthy college students (n=29, 18‐24 years old) with no prior diagnosis of a sleep disorder consented to downloading a smartphone app, the Effortless Assessment Research System (EARS) (Center for Digital Mental Health, University of Oregon; Ksana Health). For 1 week, the EARS app collected data continuously using the phone’s accelerometer, gyroscope, and exposure to light. Each morning, participants received a notification via EARS to complete a sleep diary, which asked participants what time they got into bed, fell asleep, woke up, and got out of bed. A random subset (n=13) of participants also consented to wear an ActiGraph wristwatch. All analyses examined bedtime (ie, time going to bed), risetime (ie, wake-up time), and time-in-bed (ie, duration between time-in-bed and out-of-bed) as 3 measures of interest. For all analyses, diary data were considered the reference measurement, and analyses were repeated with ActiGraph as the reference measurement.

**Results:**

On average, EARS showed a high mean true positive rate (86.6%) and low mean false positive rate (4%) based on diary-reported bedtime and risetime. Supplementary analyses comparing EARS to ActiGraph data showed a similar true positive rate (83.7%) and slightly higher false positive rate (8.5%). Although there were no significant differences in mean bedtime, risetime, and time-in-bed among sources (*P*≥.07), there was some misalignment. Compared to the diaries, EARS estimated bedtime to be later by an average of 20 minutes, risetime to be earlier by an average of 21.83 minutes, and time-in-bed to be shorter by an average of 41.82 minutes. Relative to ActiGraph estimates, EARS estimated bedtime to be earlier by an average of 21.87 minutes, but estimated risetime to be later by 2.5 minutes, and time-in-bed to be longer by 24.4 minutes. Day-to-day correlations showed that bedtimes, risetimes, and time-in-bed were positively correlated between diary and EARS (0.29≤*r*≤0.52, *P≤*.002). Similarly, day-to-day bedtimes and time-in-bed were positively correlated between ActiGraph and EARS (0.38≤*r*≤0.55, *P≤*.01), and while correlated in the expected direction, risetimes were not significantly associated between ActiGraph and EARS (*r*=0.29, *P*=.07).

**Conclusions:**

Smartphone-based sleep sensors show acceptable alignment with more established methods and may provide a feasible alternative to measuring daily sleep patterns in a scalable way. Future studies will require larger, diverse samples to corroborate findings of concordance among EARS, diary, and actigraphy data in other populations.

## Introduction

Insufficient or disrupted sleep is ubiquitous in youth and young adults. Specifically, more than 60% of college students show delayed bedtimes and wake-up times [1], which often lead to poor academic performance [2], physical and psychological health issues [3], substance use [1], depression [4], and suicidal thoughts and behaviors [5]. Given pervasive sleep alterations during the college transition, it is important to develop scalable methods to measure sleep health.

In research, daily sleep diaries (eg, via ecological sampling methodology) are often used as a primary mode of measurement of subjective sleep given their low cost and ease of administration. However, reporting on sleep every morning over extended periods of time often results in low response rates, especially in the morning relative to other times of day [6,7]. The gold standard for objectively identifying sleep problems is polysomnography, which measures brain, cardiovascular, and respiratory signals. Yet, conducting a polysomnography study can be burdensome on participants as it usually requires at least 2 sessions sleeping in a lab with intrusive equipment [8] and thus may not reflect typical sleep in the home environment. Actigraphy, typically with a wrist monitor, can measure several metrics of sleep based on motion (ie, accelerometer) and most measure ambient light. Some studies ask participants to actively press a button on the watch as an event marker indicating when they are going to bed and when they wake up. Though actigraphy is a relatively unobtrusive measurement of natural at-home sleep and has been validated against polysomnography [9], there are several challenges with implementation, including compliance (eg, adherence to wearing the watch) and battery life. Specifically, the maximum battery life for most research-grade wearables is up to ~4 weeks, which limits longer-term phenotyping. By comparison, smartphone sensors (ie, mobile accelerometry) are already integrated into participants’ lives and therefore provide a naturalistic, unobtrusive, and scalable way to monitor daily sleep health (ie, onset and duration) in real-time over longer time periods without requiring participants to integrate an extra apparatus, such as a wearable.

Smartphone sensor sleep measurement is a nascent research method that is highly scalable and has enormous potential to reach populations such as those with limited means to purchase wearables, live in difficult-to-reach places, or for whom compliance with a wearable might be low (eg, adolescents). However, there can be several limitations of leveraging smartphone technologies that were not specifically designed to measure sleep. These include difficulty detecting daytime naps and distinguishing “going-to-bed” from “going-to-sleep” and “getting-out-of-bed” from “waking up.” Nonetheless, there have been several investigations of the validity of smartphone sensor sleep measurement. In adults, across 4 smartphone apps (Sleep Cycle-Accelerometer, Sleep Cycle-Microphone, Sense, and Smart Alarm), time-in-bed estimates were significantly correlated across app and polysomnography measurements; however, other sleep features (eg, sleep efficiency and sleep staging) significantly differed from polysomnography estimates [10]. In addition, a comparison between actigraphy and the TapCounter smartphone app (QuantActions) showed a strong correlation for sleep onset and wake-up times [11] and a study of college students showed that average sleep duration (using the mindLAMP app; The Division of Digital Psychiatry at Beth Israel Deaconess Medical Center) was significantly associated with daily self-reported sleep duration and quality [12]. Together, this evidence indicates that smartphone sleep measurements may serve as a reliable proxy for estimating bedtime, risetime, and time-in-bed among healthy young adults.

This study tested whether the Effortless Assessment Research System (EARS) smartphone app [13] was a reliable method for detecting bedtime, risetime, and time-in-bed over 1 week in a sample of college undergraduates. We simultaneously collected daily diaries for self-reported sleep features. In a random subset of the sample, we also collected actigraphy via a wrist wearable, which is used as a supplementary method to compare EARS-obtained sleep features with another established research method.

## Methods

### Current Study

This study analyzed data from a broader intervention project on regularizing sleep timing [14]. We report how we determined our sample size, data exclusions, and the relevant measures for this analysis.

### Recruitment

Participants were recruited from the University of Oregon using the undergraduate psychology research participant pool, classroom announcements, and printed flyers publicly posted on campus. Participants were eligible if they were 18‐24 years old, owned an Android smartphone, had no prior diagnosis of a sleep disorder, and reported greater than 2 hours of day-to-day variability between their earliest and latest wake-up times during the past week. The latter criterion was related to a broader intervention project on regularizing sleep timing. Thirty-seven individuals were eligible and consented to participate. Two participants’ phones were incompatible with the EARS app, and 1 student withdrew from participation, resulting in a total of 34 participants who completed the study.

### Procedure

This study analyzed data from the baseline week of a 5-week microrandomized trial, as described in Latham [14]. At the consent session, participants completed questionnaires and the beta version of the EARS phone app was installed on participants’ phones. A random subset (n=18) of participants also consented to wear an ActiGraph wristwatch (ActiGraph wGT3x-BT triaxial accelerometer; ActiGraph LLC) for the week. The ActiGraph was only worn by a subset of the participants because of the limited availability of these wrist wearables.

### Power Analysis

An a priori power analysis was conducted for the broader microrandomized trial using the *MRTSampleSize* R package [15]. Thirty-three participants were required to predict the estimated effect size of the broader study (β=.3) with 80% power [14].

### Self-report

The Pittsburgh Sleep Quality Index (PSQI) [16] is a validated self-reported questionnaire that probes individuals’ typical bedtime, risetime, hours of “actual sleep,” any trouble sleeping, and sleep quality over the last month. There are 7 components, or subscales, that when combined yield a total “sleep quality” score ranging from 0‐ to 21. Higher scores indicate worse sleep quality [16].

### EARS

The EARS app was developed by the Adolescent Development and Psychopathology Team (ADAPT) at the University of Oregon to passively collect data with minimal burden to participants [13,17]. This project used the smartphone accelerometer and gyroscope data to estimate bedtime, risetime, and time-in-bed. Participants were instructed to use their phones as they normally would. They were also told to make sure that their phones do not run out of battery and to make sure there is sufficient memory storage. EARS collected data continuously, including detection of when participants’ phones were charging, moving (using the phone’s accelerometer and gyroscope), and exposed to light in lumens. EARS applied a lux threshold, such that a new lumen capture only would be recorded when the detected lumen value changed by a magnitude of 3 from the previously recorded value. Accelerometer data were collected at 10 Hz sampling rate, filtering out any changes <0.05 m/s^2^. Gyroscope data were collected at a 10 Hz sampling rate, filtering out any changes <0.01 radians per second. Data were timestamped at the level of milliseconds and then aggregated into 10-second periods. For this study, the data capture schedule for the EARS app was limited to data acquisition between 8:30 PM and 10:39 AM to reduce the burden on the device. When a sleep offset time was not detected within the available data, the algorithm defaulted to using the last available data point, which truncated times to 10:39 AM. Participants’ EARS data were encrypted and stored using Amazon Web Services [17].

### Daily Sleep Diary

Each morning, participants received a notification via EARS on their smartphone to complete a sleep diary. The daily sleep diary was based on the Pittsburgh Consensus Sleep Diary [18], and participants provided what time they got into bed (ie, “What time did you get into bed last night?”), fell asleep (ie, “What time did you fall asleep last night?”), woke up (“What time was your final awakening?”), and got out of bed (ie, “What time did you get out of bed this morning?”). Note that the EARS app went through minor changes during the study period, mainly to remove bugs.

### Actigraphy

In a supplementary part of the study, 18 participants were randomly selected and consented to wear an ActiGraph wristwatch (ActiGraph wGT3x-BT triaxial accelerometer; ActiGraph LLC) for the baseline week of the study. The Cole-Kripke algorithm was used to score sleep-wake periods [19], and the Tudor-Locke algorithm was used to detect periods of time-in-bed. We used the “In Bedtime” and “Out Bedtime” to reflect bedtime (ie, time going to bed) and risetimes (ie, wake-up time), respectively.

Out of the 34 participants who completed the study, 32 participants had usable data from at least 2 concurrent sources measured on the same day, 29 of whom had concurrent diary and EARS data, and 13 of whom also had ActiGraph data.

### Data Quality Assurance

Although actigraphy should provide 1 sleep period (excluding naps) per day, 11 participants with ActiGraph data showed more than 1 sleep period on certain days. Within each of these participants and days, we identified the earliest and latest time (ie, the longest duration) to determine the most likely sleep period and then removed entries that appeared to be midday naps given that the EARS app is not yet able to detect midday naps. We also inspected outliers upon plotting distributions of bedtime, risetime, and time-in-bed for all sources of data, which removed 10 observations that were 3 SDs above or below the mean.

### Statistical Analysis

All analyses examined bedtime (ie, time going to bed), risetime (ie, wake-up time), and time-in-bed (ie, duration between time in-bed and out-of-bed) as 3 measures of interest. For all analyses, diary data were considered the reference measurement given that (1) all participants were administered daily diaries, (2) the questions were derived from validated measures, and (3) diaries are more commonly used relative to the ActiGraph wearable used in this study and the EARS smartphone app. However, we also repeated analyses with ActiGraph as the reference measurement given that this wearable obtains accelerometry data. In addition, given that participants also self-reported onset, offset, and duration of sleep (separate from bed timings), we compared EARS estimates to these diary features in supplementary analyses.

First, we computed descriptive statistics for each source of measurement (diary, ActiGraph, and EARS). Second, we assessed the group-level true positive rates (TPR) and false positive rates (FPR) of EARS data based on minute-by-minute classification of “true” and “false” in-bed epochs. For this classification, each minute was counted as a true in-bed epoch until the next out-of-bed period for each particular data source. For the purpose of this analysis, we used the diary as the reference source. Thus, if an epoch was an in-bed period for both diary and EARS, then that minute was classified as a true in-bed epoch for EARS. Similarly, if an epoch was an out-of-bed period for both diary and EARS, then that minute was counted as a true out-of-bed period for EARS. If there was a difference or mismatch across sources (eg, diary indicated in-bed and EARS indicated out-of-bed), then that minute would be counted as a false in-bed or out-of-bed epoch for EARS. Thus, each minute of the day (1440 min) was denoted as either a true in-bed, false in-bed, true out-of-bed, or false out-of-bed period, and then aggregated at the person level (ie, for each person, we summed the true in-bed epochs, false in-bed epochs, true out-of-bed epochs, and false out-of-bed epochs). EARS TPR was computed as true in-bed epochs divided by the sum of true in-bed and false out-of-bed epochs. EARS FPR was computed as false in-bed periods divided by the sum of false in-bed and true out-of-bed periods. EARS TPR and FPR calculations were repeated based on the truncated data capture period (8:30 PM-10:39 AM; 849 min). Third, we used Bland–Altman plots (BlandAltmanLeh R package [20]) to visualize agreement between sources, where the difference between 2 sources of data is plotted against the mean of these sources. A positive value of the mean difference between diary and EARS data (Diary–EARS) indicates that EARS yielded earlier times relative to diary estimates, and a negative value means that EARS yielded later times relative to diary estimates. Next, using linear regression models, we examined whether average bedtime, risetime, and time-in-bed over the 7 days (ie, person-level) were significantly different among sources, using diary as the reference. We repeated these models with ActiGraph as the reference. Finally, we computed repeated-measures correlations between sources’ bedtime, risetime, and time-in-bed.

### Ethical Considerations

All procedures were reviewed and approved by the University of Oregon Institutional Review Board (# 08142018.013). Informed consent forms stated that the researchers planned to publish and present the results of this research and that identifying information would not be used in any published reports, conference presentations, or other presentations. Identifiable information was stored in a password-protected database that was only accessible by the researchers for the study. All other data provided were kept in separate databases. All data that the phone app collected were encrypted on the phone and kept in secure, encrypted cloud-based storage. Participants were either paid US $100 or awarded 4 credits for completing the broader intervention project.

## Results

### Descriptive statistics

**Figure 1.**
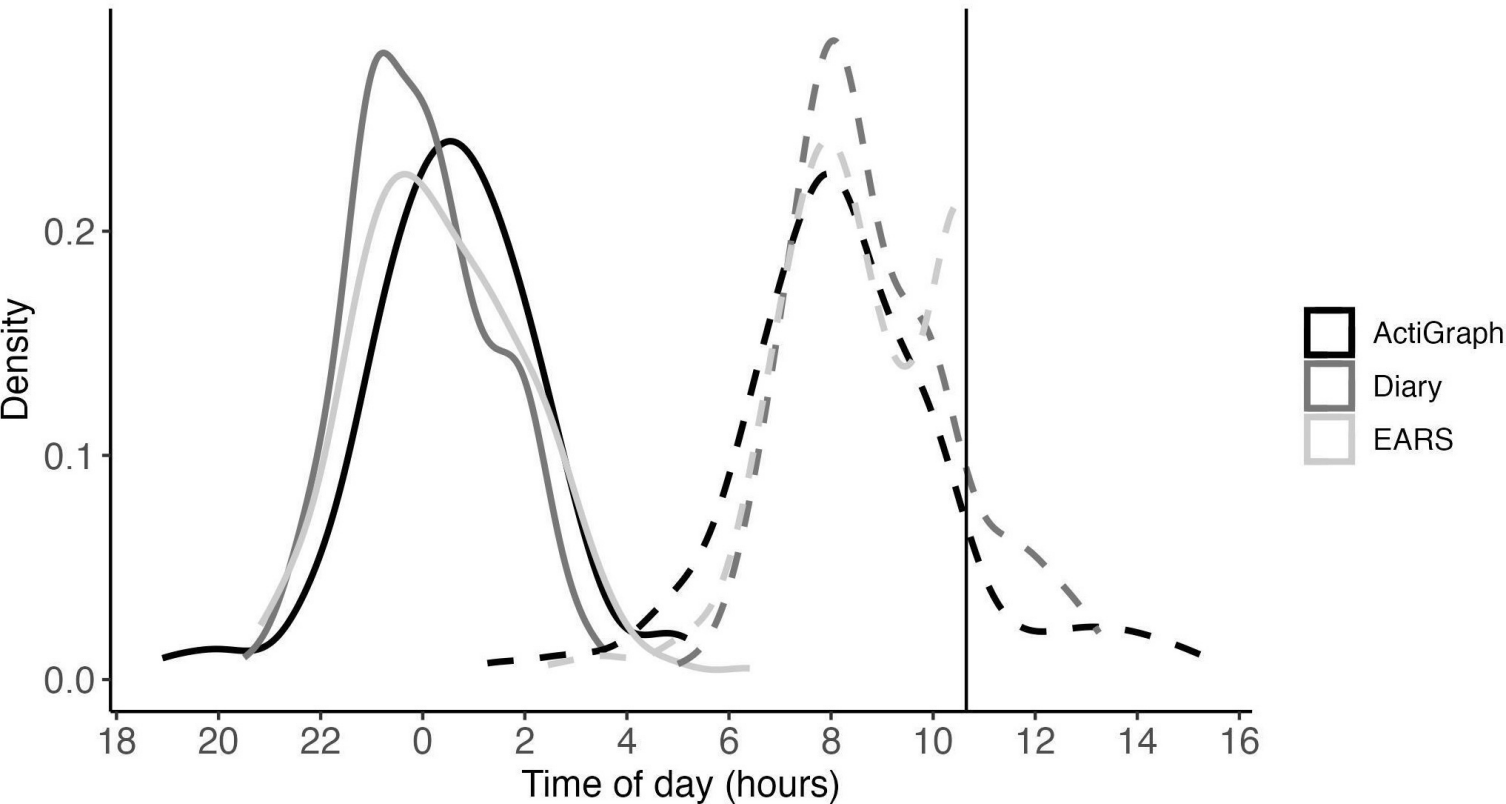
Distribution of mean bedtimes (solid lines): ActiGraph = 12:35:55 AM; Diary =11:59:54 PM; EARS=12:24:31 AM; Mean risetime (dotted lines): ActiGraph = 8:16:43 AM; Diary = 8:49:50 AM; EARS = 8:34:27 AM). The solid vertical line indicates the time at which EARS truncated data collection at 10:39 AM.

Participant demographics are presented in Table 1. Descriptive statistics for bedtime, risetime, and time-in-bed per data source (ie, diary, EARS, and ActiGraph) over the 1-week data collection period are displayed in Table 2, Figure 1, and Figure S1 in Multimedia Appendix 1. The PSQI demonstrated low internal consistency (α=.66) in this sample, but was considered for exploratory correlational analyses.

**Table 1.** Participant characteristics (n=29). Choices for biological sex were male or female.

Variable	Values
Age (years), mean (SD)	20.93 (1.86)
Sex (Female), n (%)	14 (56)
Race, n (%)	
African American or Black	2 (6.9)
Asian	3 (10.34)
More than one race	2 (6.9)
White	20 (68.97)
Unknown or Not reported	2 (6.9)
Ethnicity, n (%)	
Hispanic	1 (3.45)
Non-Hispanic	27 (93.1)
Unknown or Not reported	1 (3.45)
Number of completed days, mean (SD)	5.93 (1.69)

**Table 2.** Descriptives of bedtime, risetime, and time-in-bed.^a^

Source	Bedtime, mean (SD)	Bedtime, median (IQR)	Sleep onset, mean (SD)	Sleep onset, median (IQR)	Risetime, mean (SD)	Risetime, median (IQR)	Time in bed, mean (SD)	Time in bed, median (IQR)
Diary	–0.91 (82.64)	0 (–210 to 216)	43.37 (83.57)	30 (–180 to 300)	529.84 (97.29)	510 (300‐795)	530.74 (100.72)	513 (270‐795)
EARS	24.53 (101.27)	16.26 (–190.80 to 384.38)	—^b^	—	514.45 (101.24)	507.11 (147.2‐639.02)	489.93 (130.95)	486.03 (179.95‐829.82)
ActiGraph	35.93 (104.57)	37.50 (–306 to 310)	—	—	496.73 (139.22)	480.50 (76-914)	460.80 (156.41)	444.5 (165‐1035)

a Bedtime and risetime are represented by minutes from midnight (eg, mean diary bedtime of −0.91 corresponds to 11:59:54PM). Diary reports probed both bedtimes and sleep onset times (eg, time getting into bed and time falling asleep, respectively).

b Not applicable.

### True Positive and False Positive Rates

On average (at the group level), EARS showed a high mean TPR (86.6%, range=31.9%‐100%) and low mean FPR (4.0%, range=0.10%‐20.2%) based on diary-reported bedtime and risetime. Limiting the minute-by-minute data to the truncated data capture period for EARS (849 min per day) yielded a similar TPR (86.6%, range=31.9%‐100%) and slightly higher mean FPR (10.6%, range=0.24%‐48.6%). The TPR and FPR for EARS in-bed and out-of-bed epochs based on diary-reported sleep onset and offset show similar estimates (TPR=89.5%, range=36.3%‐100%; FPR=6.8%, range=0.80%-21.5%). Supplementary analyses comparing EARS to ActiGraph data showed a similar TPR (83.7%, range=33.1%‐100%) and similar FPR (8.5%, range=0%‐21.6%).

### Bland–Altman Agreement

**Figure 2.**
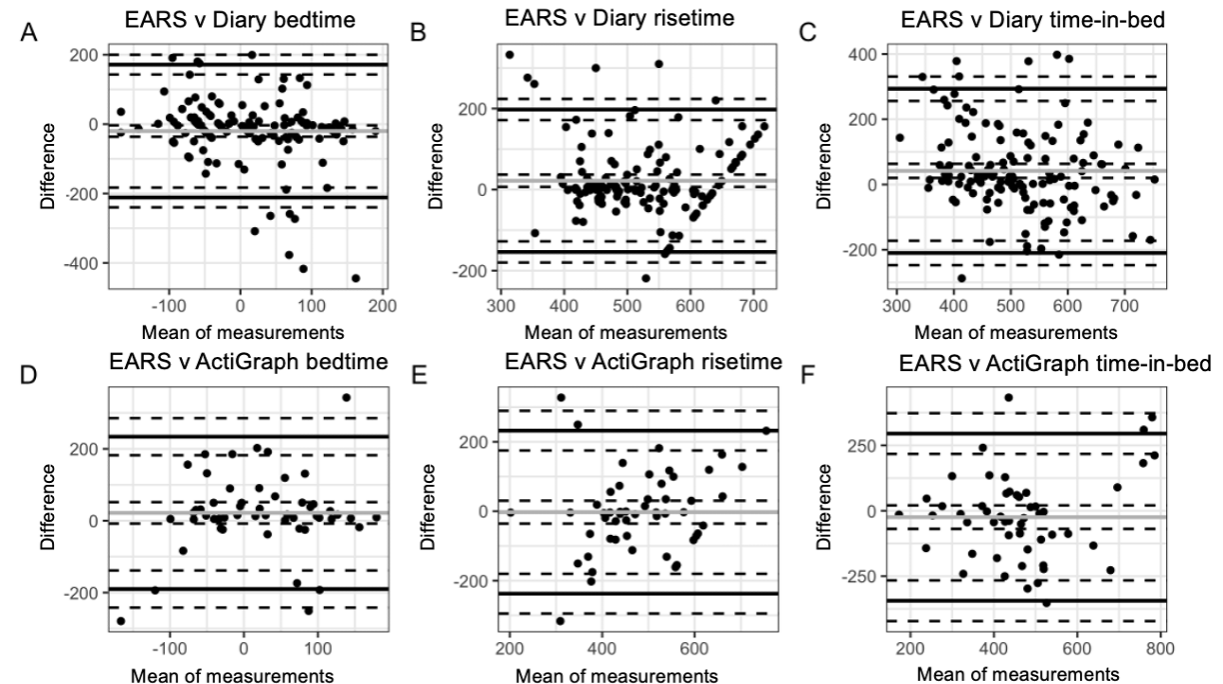
Bland-Altman plots. The x-axis represents the mean (in minutes) of 2 measurements (eg, EARS [Effortless Assessment Research System] and diary) and the y-axis represents the difference (in minutes) between the measurements. The gray solid line represents the mean difference in minutes between sources of measurement. The black solid horizontal lines indicate +1.96 SD and −1.96 SD from the mean. The black dotted lines are the CIs surrounding the mean and SD.

The majority of data showing the association between the mean of measurements (eg, averaged EARS and diary time-in-bed) and the difference between measurements (diary time-in-bed – EARS time-in-bed) were within the limits of agreement with a mean difference [SD 1.96]; Figure 2), which is in line with recommendations for adequate agreement of measures [21]. Of note, the CIs are large, indicating small sample size and relatively large dispersion of differences. Although the mean of EARS and diary measurements correlated with the difference between EARS and diary measurements for bedtime and time-in-bed (*P* ≤.01), indicating a presence of proportional bias, there was no correlation present for risetime (*P*=.80). In addition, the average of EARS and ActiGraph measurements did not correlate with the difference between EARS and ActiGraph measurements for any of the sleep features (*P* ≥.20).

### Person-level Differences in Sleep Estimates

There were no significant differences in mean bedtime, risetime, and time-in-bed between sources (*P*≥.07; Tables3^5^); however, there was some misalignment between EARS measurements relative to diary estimates. Compared to the diaries, EARS estimated bedtime to be later by an average of 20 minutes (SD 97.65), risetime to be earlier by an average of 21.83 minutes (SD 89.68), and time-in-bed to be shorter by an average of 41.82 minutes (SD 128.46) (Figure S2 in Multimedia Appendix 1). Relative to ActiGraph estimates, EARS estimated bedtime to be earlier by an average of 21.87 minutes (SD 108.15), but estimated risetime to be later by 2.5 minutes (SD 119.9) and time-in-bed to be longer by 24.4 minutes (SD 163.1) (Figure S3 in Multimedia Appendix 1). In supplementary analyses, there were no significant differences between EARS bedtime and diary sleep onset, EARS risetime and diary sleep offset, and EARS time-in-bed and diary sleep duration (Table S1 in Multimedia Appendix 1, *P*≥.07). Relative to the diaries, EARS estimated bedtime to be earlier than diary sleep onset by an average of 27.68 minutes (SD 88.89), risetime to be earlier than sleep offset by an average of 2.91 minutes (SD 91.38), and time-in-bed to be longer than sleep duration by an average of 24.77 minutes (SD 126.51) (Figure S4 in Multimedia Appendix 1). Individual plots showing overlap of bedtime and risetime across sources are shown in Figure S5 in Multimedia Appendix 1.

**Table 3.** Comparing Effortless Assessment Research System and diary bedtime, risetime, and time-in-bed (n=29). The intercept reflects Diary as the reference group.

Sleep features	b	95% CI	*t* test^a^	*P* value
Bedtime				
Intercept	−0.73	−23.94 to 22.47	−0.06	0.95
Source (EARS)^b^	18.38	−14.44 to 51.19	1.12	0.27
Risetime				
Intercept	534.97	507.96 to 561.98	39.67	<.001
Source (EARS)	−8.17	−46.37 to 30.03	−0.43	0.67
Time-in-bed				
Intercept	535.7	[504.02 to 567.38]	33.88	<.001
Source (EARS)	−26.55	[−71.35 to 18.25]	−1.19	0.24

a Degrees of freedom (df)=56.

b EARS: Effortless Assessment Research System.

**Table 4.** Comparing Effortless Assessment Research System and diary bedtime, risetime, and time-in-bed to Actigraph bedtime, risetime, and time-in-bed (n=13). The intercept reflects ActiGraph as the reference group.

Sleep features	b	95% CI	*t* test^a^	*P* value
Bedtime				
Intercept	37.12	5.66 to 68.57	2.38	0.02
Source (Diary)	−41.14	−85.62 to 3.35	−1.86	0.07
Source (EARS)^b^	−42.20	−88.24 to 3.84	−1.85	0.07
Risetime				
Intercept	502.77	459.01 to 546.52	23.17	<.001
Source (Diary)	8.69	−53.19 to 70.58	0.28	0.78
Source (EARS)	12.77	−51.28 to 76.83	0.40	0.69
Time-in-bed				
Intercept	465.65	416.25 to 515.05	19.01	<.001
Source (Diary)	49.83	−19.97 to 119.63	1.44	0.16
Source (EARS)	54.97	−17.27 to 127.22	1.53	0.13

a Degrees of freedom (*df*)=43.

b EARS: Effortless Assessment Research System.

**Table 5.** Comparing Effortless Assessment Research System and ActiGraph bedtime, risetime, and time-in-bed to diary sleep features (n=13). The intercept reflects Diary as the reference group. The intercept is different compared to the intercept in Table 3 because this table represents a subset of those individuals who wore the ActiGraph.

Sleep features	b	95% CI	*t* test^a^	*P* value
Bedtime				
Intercept	−4.02	−35.47 to 27.44	−0.26	0.80
Source (ActiGraph)	41.14	−3.35 to 85.62	1.86	0.07
Source (EARS)^b^	−1.07	−47.11 to 44.98	−0.05	0.96
Risetime				
Intercept	511.46	467.70 to 555.22	23.57	<.001
Source (ActiGraph)	−8.69	−70.58 to 53.19	−0.28	0.78
Source (EARS)	4.08	−59.98 to 68.13	0.13	0.90
Time-in-bed				
Intercept	515.48	466.12 to 564.83	21.06	<.001
Source (ActiGraph)	−49.83	−119.63 to 19.97	−1.44	0.16
Source (EARS)	5.15	−67.10 to 77.39	0.14	0.89

a Degrees of freedom (*df*)=43.

b EARS: Effortless Assessment Research System.

### Cross-modality Correlations

**Figure 3.**
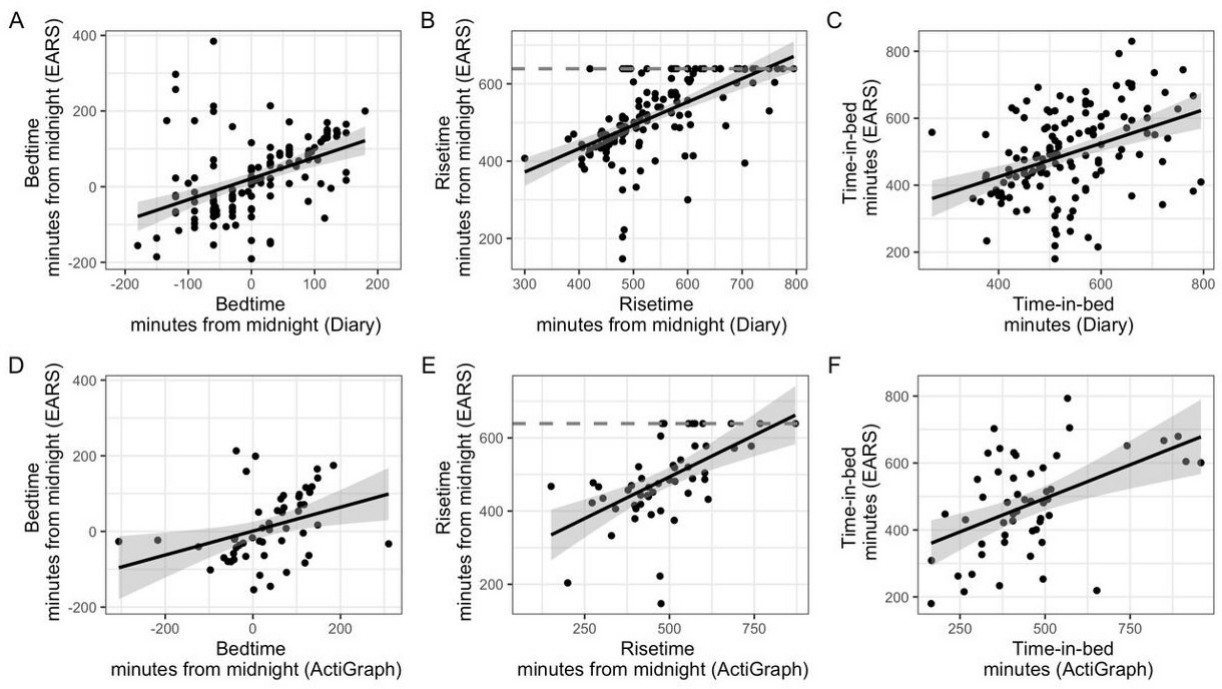
Correlations among diary, Effortless Assessment Research System, and ActiGraph sleep features. The top panel depicts associations between Diary and Effortless Assessment Research System, and the bottom panel shows associations between ActiGraph and Effortless Assessment Research System data. The gray dashed line in plots B and E indicates the time at which EARS truncated data collection at 639 minutes from midnight. EARS: Effortless Assessment Research System.

As shown in Figure 3, day-to-day bedtimes, risetimes, and time-in-bed were significantly positively correlated between diary and EARS (0.52≤r≥0.29, *P*≤.002). Similarly, day-to-day bedtimes and time-in-bed were significantly positively correlated between ActiGraph and EARS (0.55≤r≥0.38, *P*≤.01), and while trending in the expected direction, risetimes were not correlated between ActiGraph and EARS (*r*=0.29, *P*=.07). EARS-based bedtimes, risetimes, and time-in-bed were also significantly correlated with diary sleep onset, offset, and sleep duration (0.48≤ r≥0.44, *P*≤.001; Figure S6 in Multimedia Appendix 1). As an exploratory analysis, we correlated person-level EARS and PSQI bedtime, risetime, and time-in-bed and found no significant correlation between these sources of measurement (0.20≤ r≥−0.001, *P*≥.31). 

## Discussion

### Principal Findings

This study tested the concordance among smartphone-based, diary, and wearable (ie, actigraphy) sources of data to measure common metrics of sleep health. We found that when comparing diary and EARS in-bed and out-of-bed periods across 24 hours, EARS yielded a high TPR and low FPR. Given the EARS data capture was scheduled for 849 minutes of the day, we repeated these analyses, finding a similar TPR, but slightly higher FPR which could indicate that motion-based sleep measurement is a slightly more sensitive measure than diary-reported measurement, particularly if most people have their phones with them in-bed or near the bed. Notably, EARS TPR and FPR based on ActiGraph in-bed and out-of-bed epochs were similar to estimates yielded from diary and EARS alignment. In addition, Bland–Altman visualizations revealed adequate agreement of measures. However, upon inspection of the correlation between the mean of measures and the difference between measures, we found evidence of proportional bias specifically for EARS bedtimes and time-in-bed relative to diary bedtimes and time-in-bed, which could indicate that EARS measurement error varies with bedtimes and time spent in bed. Alternatively, as this bias was not present when comparing EARS and ActiGraph estimates for any of the sleep features, the correlation may indicate misestimation from diary data.

On average, there was no significant difference between sources in bedtime, risetime, or time-in-bed estimates. In addition, there was no significant difference between diary-reported sleep onset, offset, and duration and EARS-based bedtime, risetime, and time-in-bed; however, there was some misalignment. EARS tended to estimate bedtime to be later compared to diary-reported bedtime, but earlier relative to ActiGraph bedtime and diary-reported sleep onset. This misalignment could reflect people putting their phones down later compared to when they report initially going to bed. In addition, EARS tended to estimate earlier risetimes relative to diary-reported risetimes and diary-reported sleep offset times, but estimated slightly later risetimes (~2.5 min) relative to ActiGraph risetimes. It is likely that people are picking up their phones in bed when EARS detects “risetime” prior to getting out of bed, which is when they are self-reporting their risetimes. EARS also estimated a shorter time-in-bed compared to diary-reported time-in-bed, which is a result of EARS estimating a later bedtime and earlier risetime. Taken together, it is not surprising that different forms of assessments (self-report, wrist accelerometry, and smartphone sensors) yield discrepant estimates in bedtime, risetime, and time-in-bed as each of these methods relies on different environmental and internal factors (eg, perception of sleep when self-reporting, light and movement for accelerometry, and usage of smartphones during bedtime and risetime).

Repeated correlation analyses revealed that EARS bedtime, risetime, and time-in-bed estimates were significantly correlated with daily diary and actigraphy measurements, except for the correlation between ActiGraph and EARS risetimes, which trended in the expected direction. It is possible that the low number of participants completing the ActiGraph portion of the study diminished our ability to detect a stronger correlation. In addition, the association between PSQI estimates and EARS estimates of bedtime, risetime, and time-in-bed was not associated. There are several reasons that could account for this lack of association, including the difference in acquisition of sleep estimates (ie, self-report with the PSQI compared to passive sensing with EARS) and the timescale of reporting. First, self-reported sleep estimates may be affected by perceived sleep quality and passive sensing estimates may be affected by individual differences in phone usage. Second, the PSQI aims to estimate sleep features over the course of the past month, whereas EARS obtains estimates of sleep features in real time. However, over the course of a week, we found that EARS yielded sleep health estimates that were adequately concordant with self-reported daily diary and ActiGraph estimates in healthy young adults.

Research regarding smartphone-based sleep health is still emerging. Our findings are consistent with other research on college students showing alignment between smartphone-based and self-reported sleep duration [12], onset, and wake-up times [22]. Other healthy adult samples show associations between smartphone and actigraphy onset and wake-up times [11], and total sleep time [23].

Although we show adequate concordance among bedtimes, risetimes, and duration of time-in-bed in this young adult college sample, prior research in young adult samples has shown misalignment in other sleep features (eg, sleep efficiency, sleep latency, and deep sleep) obtained through mobile apps compared to polysomnography, except for time-in-bed [10]. It is possible that smartphone apps may not yet have the capability to accurately measure complex sleep features (eg, efficiency, latency, and sleep cycle stages). Our findings also conflict with research of younger developmental age groups with disordered sleep. Children with sleep-disordered breathing showed significant differences between smartphone-based sleep metrics (sleep onset latency, total sleep time, wake after sleep onset, and sleep efficiency) and polysomnography [24]. Therefore, mobile sensors may be limited in detecting subtle sleep movements, particularly in populations with disordered sleep.

There are several limitations to note. First, although there is concordance among EARS, diary, and ActiGraph sleep features, the small sample size makes it challenging to robustly determine the reliability and validity of EARS. Second, our sample was healthy, enrolled in college, mostly White, and female, which may not generalize our findings to other populations. In particular, college students may experience daily irregularities in their sleep schedule, as many are experiencing autonomy for the first time and balancing academic and social needs [25]. Finally, we assessed sleep over a week, which limits our ability to determine whether alignment of mobile sensors, diary, and actigraphy potentially differs over longer periods of time.

### Conclusions

Taken together, smartphone sensors appear to be reliable at detecting basic features of sleep health (ie, bedtime, risetime, and time-in-bed), although whether the level of reliability is adequate will depend on the use case. One of the potential uses of smartphone data is to collect data on sleep at larger scales, and for such apps, the tradeoff between accuracy and scalability may be justified. For example, smartphone methods may be less appropriate in applicants where very precise measurements of sleep timing are critical. Furthermore, although these data may be informative for detecting alterations in sleep behavior that may vary within individuals over time, smartphone methods are likely to be limited in detecting other features that require increased sensitivity to subtle movements (although these were not examined here). Although this feasibility study shows promise in the alignment across passive (smartphone accelerometer and wrist accelerometer) and self-report (daily diary) measurements, objective and subjective sleep measurements can often differ. Several considerations, including sample size, age range of population, cost, clinical acuity of the population, and goal of the study (eg, perceived sleep and objective sleep) are factors that may impact the choice of sleep measurement. Future studies will require larger and more diverse samples to corroborate our findings of concordance among EARS, diary, and actigraphy data. As noted above, given the feasibility of using smartphones in the general population, our findings show preliminary evidence for using mobile sensors as a scalable method to detect sleep health behaviors.

## Supplementary material

10.2196/67455 Multimedia Appendix 1 Differences in sleep features and their distribution patterns based on sources.

## Abbreviations

EARS: Effortless Assessment of Research System
FPR: false positive rate
PSG: polysomnography
PSQI: Pittsburgh Sleep Quality Index
TPR: true positive rate
