# Supplementary material for: Comparison of Sleep Features Across Smartphone Sensors, Actigraphy, and Diaries Among Young Adults: Longitudinal Observational Study
Source: JMIR Form Res. 2025 Aug 14;9:e67455. doi: 10.2196/67455 (PMC12352797; doi:10.2196/67455)
Supplement: Multimedia Appendix 1 [file formative-v9-e67455-s001.docx]

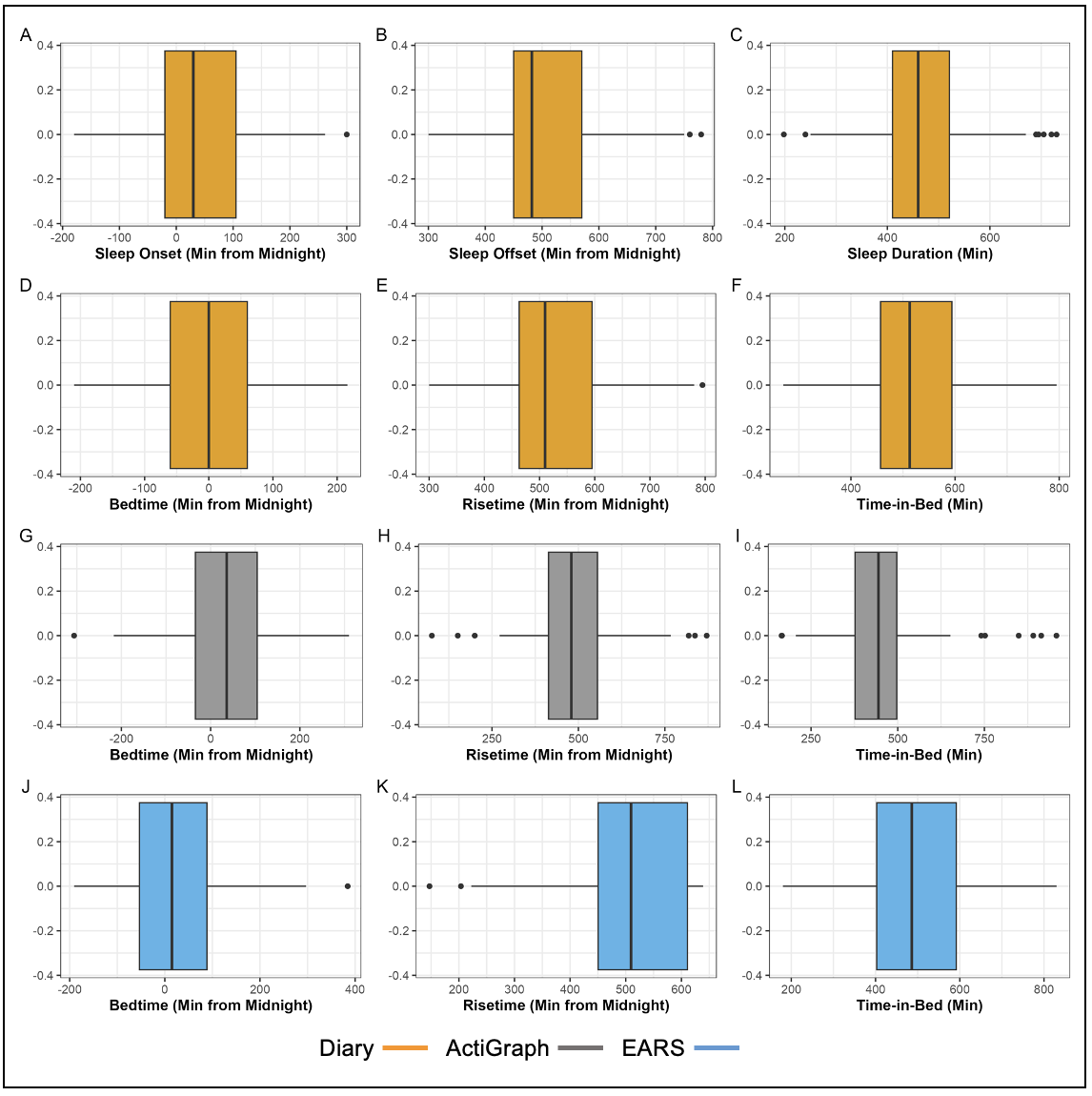


**Figure S1.** Distribution of sleep features observed through the daily diary, ActiGraph, and EARS.


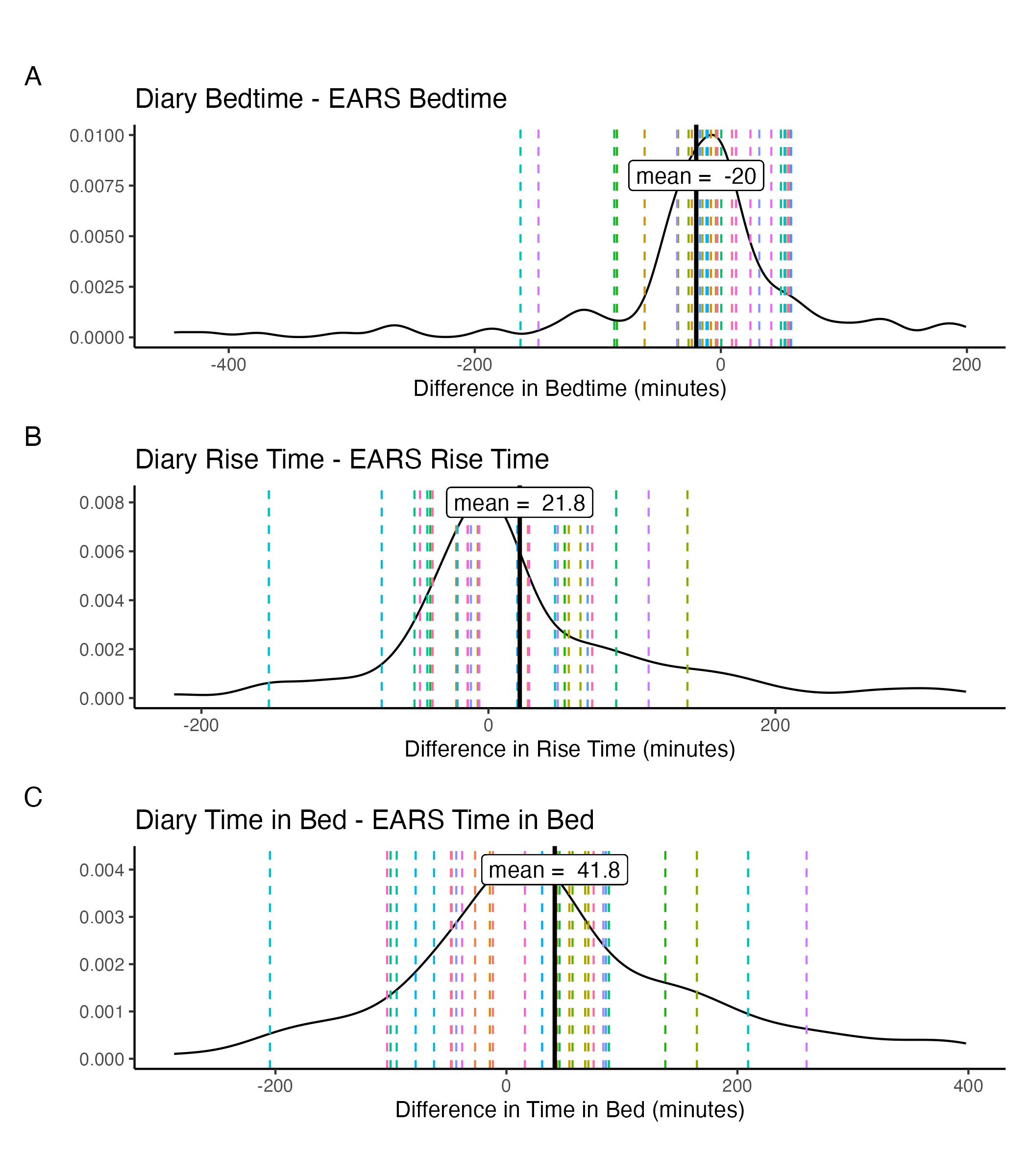


**Figure S2.** Distributions reflect the difference (in minutes) among (A) Diary and EARS bedtime (minutes from midnight), (B) Diary and EARS risetime (minutes from midnight), and (C) Diary and EARS time in bed. The colored dotted lines represent each person’s mean difference. The solid black vertical line with the mean displayed represents the group average difference across days. A positive mean indicates EARS underestimated the time relative to Diary estimates, and a negative mean indicates that EARS overestimated the time relative to Diary estimates.

**
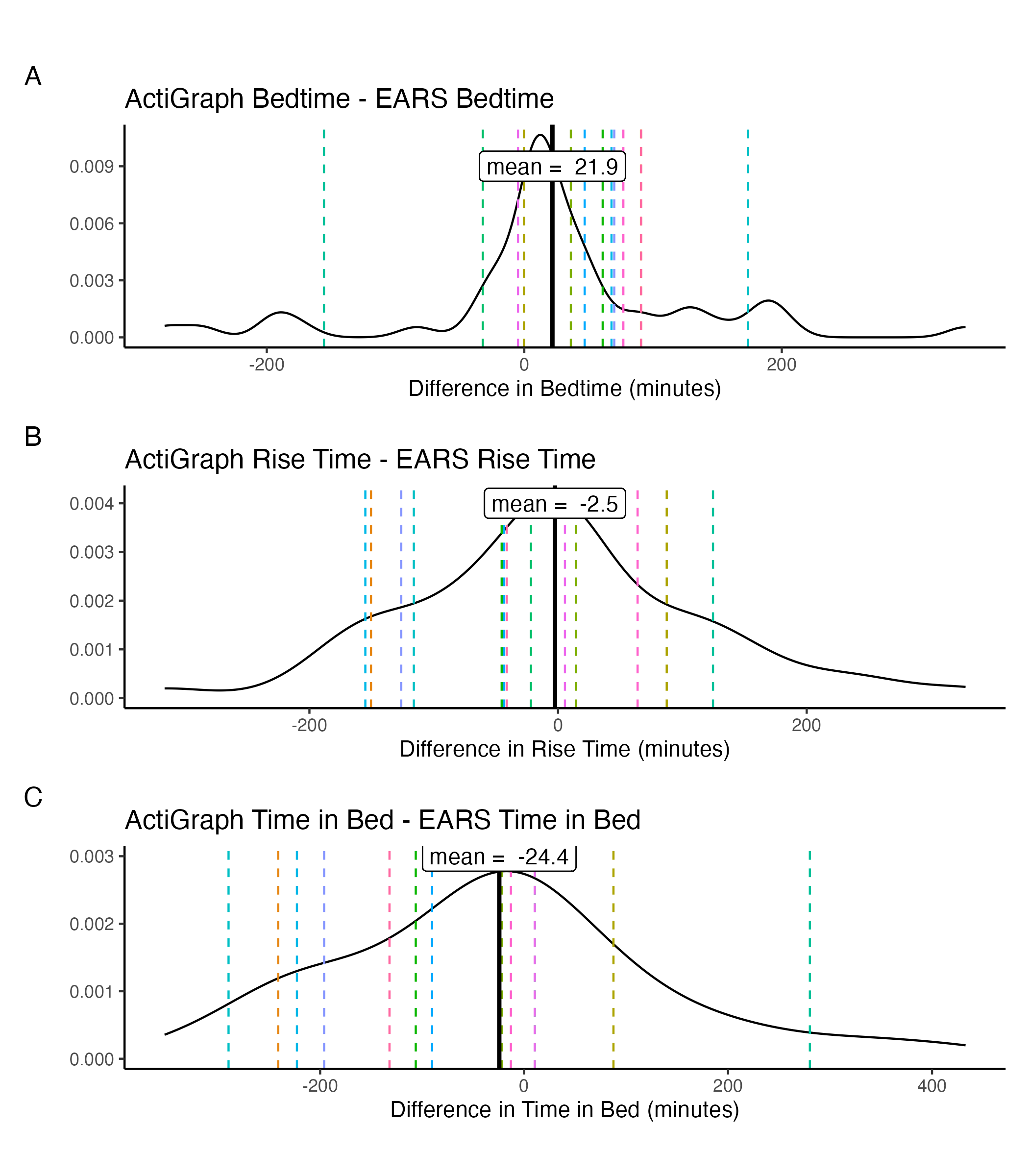
**

**Figure S3.** Distributions reflect the difference (in minutes) among (A) ActiGraph and EARS bedtime (minutes from midnight), (B) ActiGraph and EARS risetime (minutes from midnight), and (C) ActiGraph and EARS time-in-bed. The colored dotted lines each person’s mean difference. The solid black vertical line with the mean displayed represents the group average difference across days. A positive mean indicates EARS underestimated the time relative to ActiGraph estimates, and a negative mean indicates that EARS overestimated the time relative to ActiGraph estimates.

| **Table S1. Comparing EARS Bedtime, Risetime, and Time-in-Bed to Diary Sleep Onset, Offset, and Duration (N=29)** | | | | |
| --- | --- | --- | --- | --- |
|  | **𝑏** | **95% CI** | **𝑡** | **𝑝** |
| **Bedtime/Sleep Onset** |  |  |  |  |
| Intercept | 47.54 | [23.16, 71.92] | 3.91 | <0.001 |
| Source [EARS] | -29.9 | [-64.38, 4.58] | -1.74 | 0.088 |
| **Risetime/Sleep Offset** |  |  |  |  |
| Intercept | 516.12 | [488.94, 543.31] | 38.03 | < .001 |
| Source [EARS] | 10.68 | [-27.77, 49.12] | 0.56 | 0.580 |
| **Time-in-Bed/Sleep Duration** |  |  |  |  |
| Intercept | 468.58 | [438.00, 499.16] | 30.70 | < .001 |
| Source [EARS] | 40.58 | [-2.67, 83.82] | 1.88 | 0.065 |
| *Note.* The intercept reflects Diary as the reference group. Degrees of freedom=56 | | | | |

**
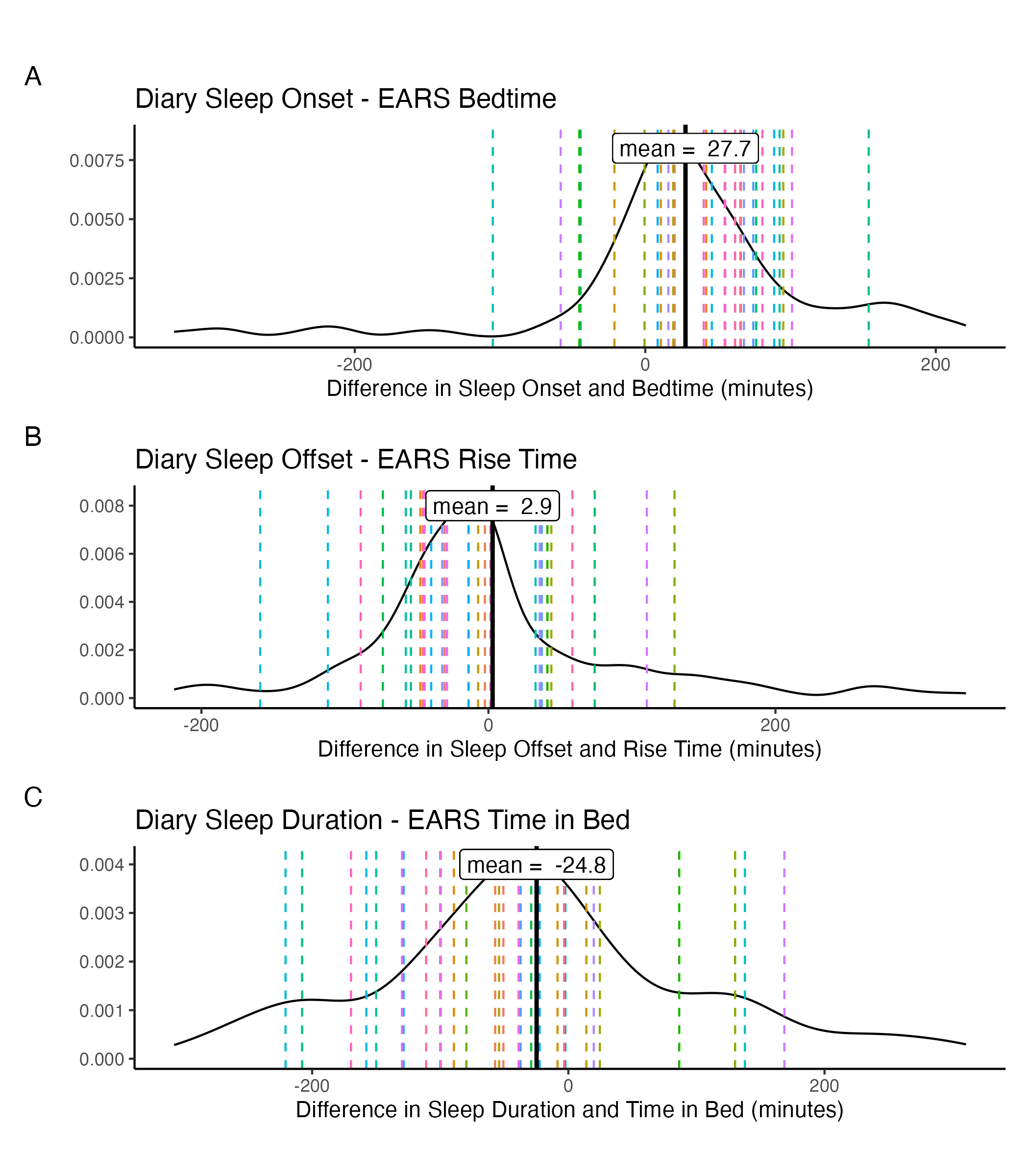
**

**Figure S4.** Distributions reflect the difference (in minutes) among (A) Diary sleep onset and EARS bedtime (minutes from midnight), (B) Diary sleep offset and EARS risetime (minutes from midnight), and (C) Diary sleep duration and EARS time-in-bed. The colored dotted lines represent each person’s mean difference. The solid black vertical line with the mean displayed represents the group average difference across days. A positive mean indicates EARS underestimated the time relative to Diary estimates, and a negative mean indicates that EARS overestimated the time relative to Diary estimates.

**
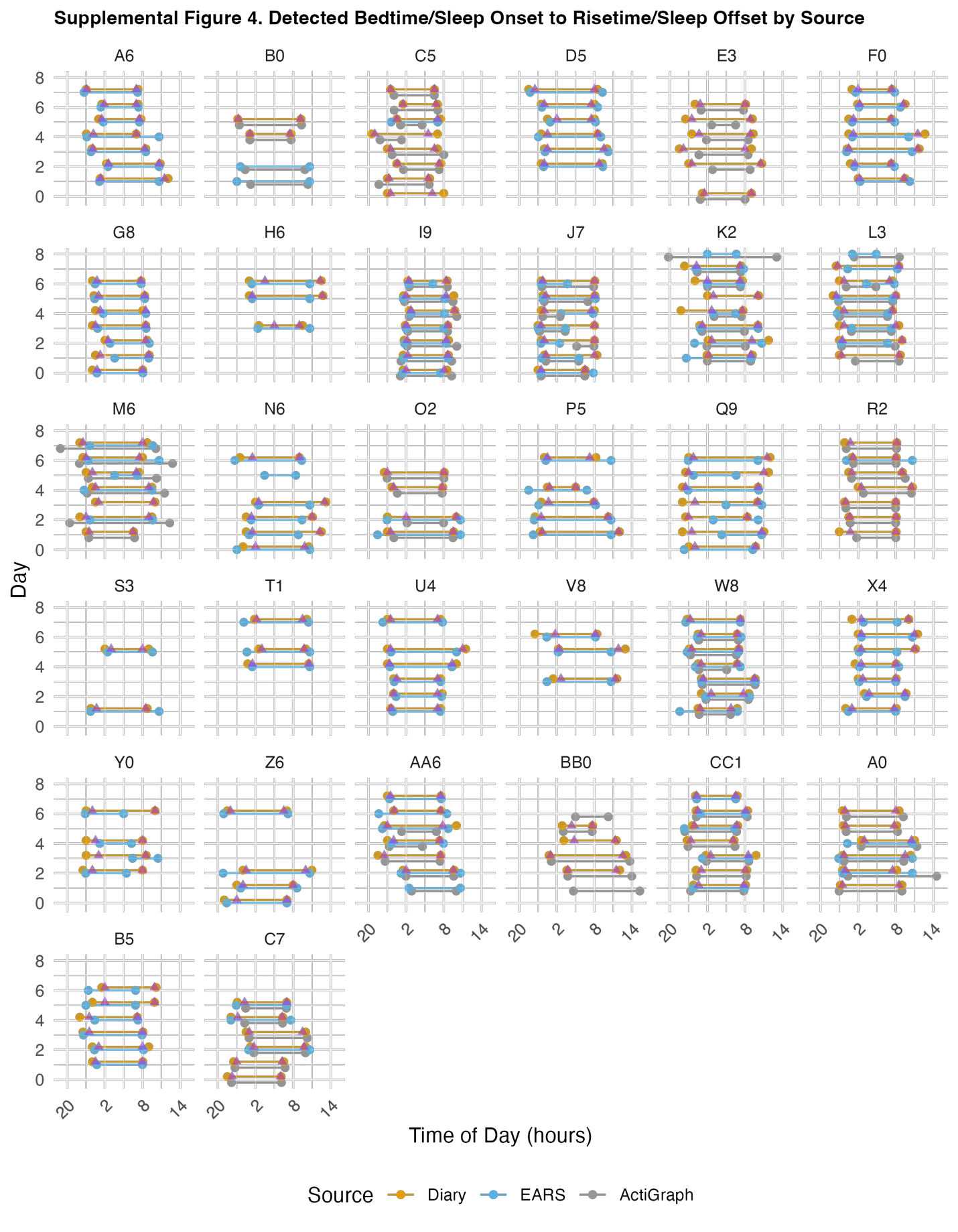
Figure S5.** Participant-level data showing bedtime and rise times (x-axis) by day (y-axis) and source (Diary, EARS, ActiGraph). Purple triangles indicate diary reported sleep onset and offset times.


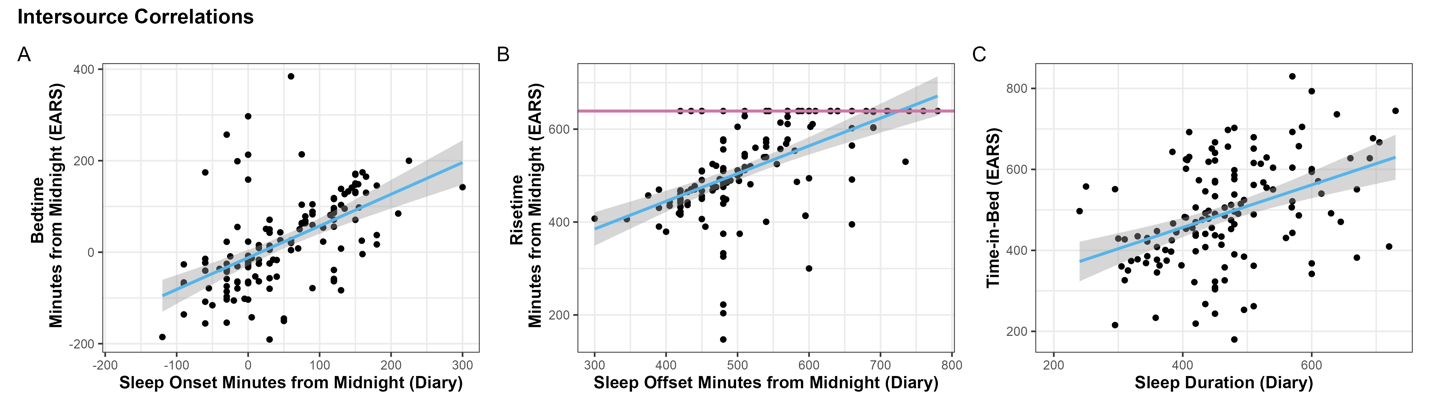


**Figure S6.** Correlations between (A) Diary sleep onset and EARS bedtime, (B) Diary sleep offset and EARS risetime, and (C) Diary sleep duration and EARS time-in-bed. The purple solid line in plots B and E indicates the time at which EARS truncated data collection at 639 minutes from midnight.
